# GmNAC5, a NAC Transcription Factor, Is a Transient Response Regulator Induced by Abiotic Stress in Soybean

**DOI:** 10.1155/2013/768972

**Published:** 2013-07-28

**Authors:** Hangxia Jin, Guangli Xu, Qingchang Meng, Fang Huang, Deyue Yu

**Affiliations:** State Key Laboratory of Crop Genetics and Germplasm Enhancement, National Center for Soybean Improvement, Nanjing Agricultural University, Nanjing 210095, China

## Abstract

GmNAC5 is a member of NAM subfamily belonging to NAC transcription factors in soybean (*Glycine max *(L.) Merr.). Studies on NAC transcription factors have shown that this family functioned in the regulation of shoot apical meristem (SAM), hormone signalling, and stress responses. In this study, we examined the expression levels of *GmNAC5*. *GmNAC5* was highly expressed in the roots and immature seeds, especially strongly in immature seeds of 40 days after flowering. In addition, we found that *GmNAC5* was induced by mechanical wounding, high salinity, and cold treatments but was not induced by abscisic acid (ABA). The subcellular localization assay suggested that GmNAC5 was targeted at nucleus. Together, it was suggested that GmNAC5 might be involved in seed development and abiotic stress responses in soybean.

## 1. Introduction

Environmental stresses such as drought, salinity, and cold are major factors that significantly limit agricultural productivity. NAC transcription factors play essential roles in response to various abiotic stresses [1]. The N-terminal region of NAC proteins contains a highly conserved NAC domain, which can be divided into five subdomains based on sequence similarities and may function as DNA-binding region. The C-terminal regions of NAC proteins, which exhibit the transactivation activity, are highly divergent in both sequence and length [2–4]. This family of transcription factors is involved in a lot of plant developmental processes, including shoot apical meristem formation [5], hormone signaling [2, 6], regulation of cell division and cell expansion [7], control of secondary wall formation [8–10], and responses to various stresses [11–14].

The NAC family consists of several subfamilies [15]. The NAM subfamily is the best studied NAC subfamily. *CUC1* and *CUC2*, encoding NAM subfamily proteins, are a pair of functionally redundant genes, expressed in *Arabidopsis* meristem and organ primordia boundary [1, 16]. The cotyledons of the transgenic seedlings overexpressing *CUC1* (*35S::CUC1*) regularly had two basal lobes, small and round epidermal cells between the sinuses, and adventitious SAMs on the adaxial surface of this region [17]. It has been reported that CUC2 is essential for dissecting the leaves of a wide range of lobed/serrated *Arabidopsis* lines. Inactivation of *CUC3 *leads to a partial suppression of the serrations, indicating a role for this gene in leaf shaping. Morphometric analysis of leaf development and genetic analysis provide evidences for different temporal contributions of CUC2 and CUC3 [18]. The *CUP* played an important role in the lateral organ boundary forming snapdragon. Cupuliformis mutants are defective in shoot apical meristem formation, but cup plants overcome this early barrier to development to reach maturity. *CUP *encodes a NAM protein, homologous to the petunia NAM and *Arabidopsis* CUC proteins. The phenotype of *cup* mutants differs from the phenotype of *NAM* and *CUC1 CUC2* in that dramatic organ fusion is observed throughout development [19]. Phloem transport of *CmNACP* mRNA was proved directly by heterograft studies between pumpkin and cucumber plants, in which *CmNACP* transcripts were shown to accumulate in cucumber scion phloem and apical tissues [20]. Petunia NAM proteins were mainly expressed in the meristem and primordia boundaries, which might be required by embryo and flower pattern formation [5]. For abiotic stress, it was observed that *Arabidopsis AtNAC2* expression was induced by salt stress and this induction was reduced in magnitude in the transgenic *Arabidopsis* plants overexpressing tobacco ethylene receptor gene *NTHK1. AtNAC2* was localized in the nucleus and had transcriptional activation activity. It can form a homodimer in yeast. *AtNAC2* was highly expressed in roots and flowers but less expressed in other organs examined. In addition to the salt induction, *AtNAC2* can be induced by abscisic acid (ABA), ACC, and NAA [21]. These showed that the NAM subfamily members not only play a regulatory role in plant development but also participate in stress responses. *GmNAC5*, which is a member of NAM subfamily belonging to NAC transcription factor in soybean, was cloned and analysed [22]. In order to further study the physiological and biochemical processes that* GmNAC5* gene may be involved in, the soybean organ expression patterns of the gene and the relationship between *GmNAC5* gene and abiotic stress were examined.

## 2. Materials and Methods

### 2.1. Plant Materials

Soybean cv. Ludou 10th was used in this study. Plants were field-grown under normal conditions in Nanjing Agricultural University. Vegetable tissues such as roots, stems, and leaves were collected from 4-week-old seedlings, while floral buds at R1 stage [23], young pods at R3 stage, and developing seeds from 15 to 50 days after flowering (DAF) were collected and frozen immediately in liquid nitrogen and stored at −80°C until use. 

### 2.2. RNA Isolation, cDNA Synthesis, and Quantitative Real-Time PCR

Total RNA was extracted using a Total RNA Plant Extraction Kit (Tiangen, Beijing, China), according to the manufacturer's protocol. First-strand cDNA was synthesized using the TaKaRa PrimeScript 1st strand cDNA Synthesis Kit (TaKaRa, Dalian, China), according to the manufacturer's instructions. Quantitative real-time polymerase chain reaction (qRT-PCR) was conducted using the SYBR Green Real-Time PCR Master Mix (TOYOBO, Osaka, Japan) on an ABI7500 Real-Time PCR System (Applied Biosystems, Foster City, CA, USA). Gene expression was quantified using the comparative method Ct: 2^−ΔΔCt^ method as previously described [24]. 

### 2.3. Stress Treatments

The soybean seedlings cultured with sand were moved to Hoagland nutrient solution, when growing to two true leaves. After the first cluster of fronds grew, the plants were applied with stress treatments with three replicates. For hormone treatments, the seedlings were treated with 100 *μ*M JA and 100 *μ*M of ABA, respectively. For salt stress, the seedlings were treated with 200 mM NaCl. For dehydration stress, the seedlings were placed on filter paper, respectively. For cold stress, the seedlings were placed in 4°C light incubator. For mechanical wounding, the seedling leaves were cut into pieces with a sharp and clean scissor. After each treatment, the leaves were harvested and frozen in liquid nitrogen immediately.

### 2.4. Subcellular Localization of NAC Proteins

The full-length cDNA of *GmNAC5* was cloned in pBI121-GFP vector, in frame fusing with GFP reporter gene and producing the plasmid pBI-GmNAC5-GFP. After transient expression of the fusion plasmid in onion epidermal cells, the cells were observed under florescence microscope. 

## 3. Results 

### 3.1. Genomic Structure of *GmNAC5*

**Figure 1:**
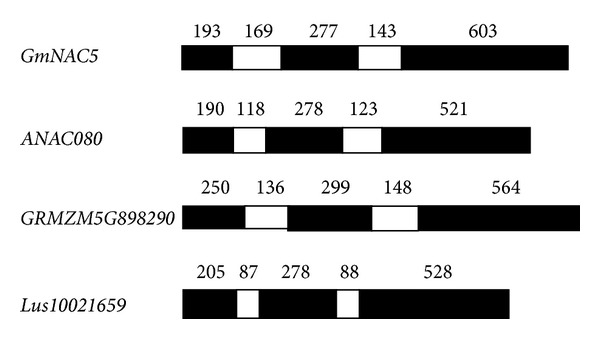
Schematic diagram of gene structures of *GmNAC5* and its homologous genes in *Arabidopsis thaliana *(*ANAC08)*, *Zea mays *(*GRMZM5G898290), *and *Linum usitatissimum *(*Lus10021659*). The black and white boxes indicate exons and introns, respectively.

NAC transcription factors have been considered one of the largest families of transcription factors so far discovered in the plant genomes. *GmNAC5* encodes a NAC transcription factor belonging to the NAM subfamily. It was found that the exon-intron structures were conserved among *GmNAC5* homologous genes in three common species, including* Arabidopsis thaliana*, *Zea mays,* and *Linum usitatissimum* (Figure 1).

### 3.2. Subcellular Localization

**Figure 2:**
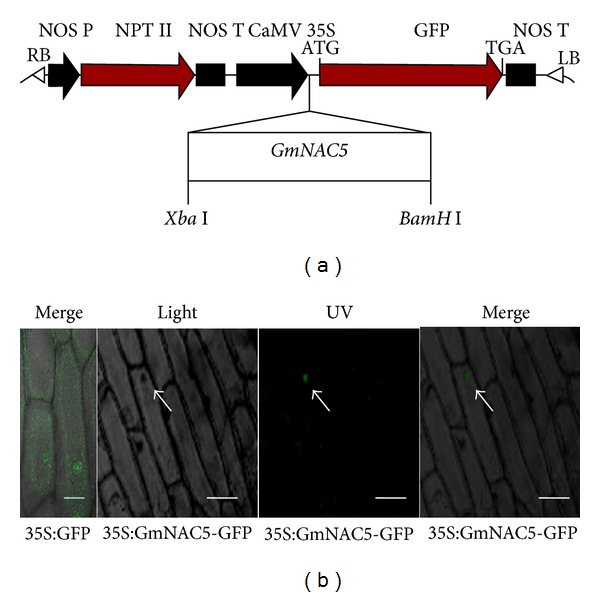
Subcellular localization of GmNAC5. (a) The structure of 35S:GmNAC5-GFP vector. (b) Subcellular localization of GmNAC5-GFP fusion protein. The arrow indicates the location of the nucleus. Bars: 40 *μ*m in 35S:GFP; 80 *μ*m in 35S:GmNAC5-GFP.

*GmNAC5 *encoding product is presumed to act as a transcription factor. If transcription factors achieve the precise adjustment of the target genes, this specific transcription factor should be located in the nucleus. Interestingly, GmNAC5 lacks the traditional nuclear localization signal (NLS); even some researchers have found that some NAC domain proteins have the nuclear localization signals [16, 25, 26]. To clarify whether soybean NAC protein GmNAC5 is located in the nucleus, the subcellular localization assay was performed (Figure 2). Despite the transient expression in the onion epidermal cells, it was observed that the GmNAC5-GFP fusion protein was located predominantly in the nucleus whereas GFP alone was localized throughout the cells (Figure 2(b)). 

### 3.3. Tissue-Specific Expression of *GmNAC5*

**Figure 3:**
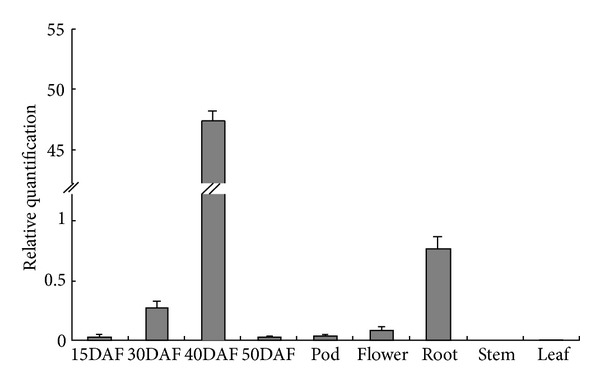
Real-time RT-PCR analysis of *GmNAC5* expression in various soybean tissues. DAF: days after flowering.

In order to analyze the physiological and biochemical processes that* GmNAC5* gene may involve, qRT-PCR approach was used to analyze *GmNAC5* gene expression in soybean in different tissues and organs. *GmNAC5* was mainly expressed in the roots and seeds in soybean development and weakly expressed in the other organs (Figure 3).* GmNAC5* has the lowest expression level in the stems, but the highest expression level in soybean seeds of 40 days after flowering. The difference in *GmNAC5* expression level of each period in soybean seed development is obvious. The highest expression level was found 40 days after flowering (DAF), but only weak expression in the seeds of 15 days and 50 days after flowering, which indicates that the *GmNAC5* may participate in the middle stage of soybean seed development. We found that *GmNAC5 *has strong expression in roots, but expression levels in stems, leaves, and pods are weak.

### 3.4. Expression of *GmNAC5* in Soybean under Various Stresses

**Figure 4:**
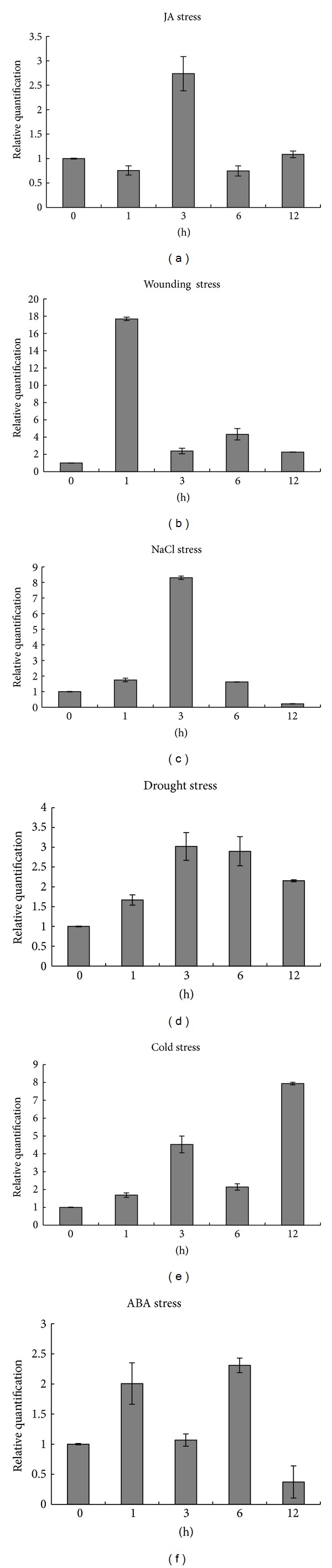
Expression of *GmNAC5* in soybean seedlings under various stresses. The soybean seedlings were stressed with 100 *μ*M JA (a), wounding (b), 200 mM NaCl (c), drought (d), 4°C (e), and 100 *μ*M ABA (f).

*GmNAC5* was weakly expressed in leaves in soybean under normal growth condition. The real-time qRT-PCR was performed to detect the expression of *GmNAC5* in soybean under various stresses (Figure 4). For jasmonic acid treatment, *GmNAC5* was significantly induced after 3 h of JA treatment (Figure 4(a)). For mechanical wounding, expression of *GmNAC5 *was sharply induced after 1 h of treatment (Figure 4(b)). For NaCl treatment, *GmNAC5* expression was markedly upregulated by 8-fold after 3 h of treatment and then decreased (Figure 4(c)). Under drought treatment, expression of *GmNAC5 *showed a weak increase and then declined (Figure 4(d)). For cold stress, it was found that *GmNAC5* expression was gradually increased and reached the maximum after 12 h of treatment (Figure 4(e)). In order to reveal whether stress responsive expression of *GmNAC5* was involved in ABA pathway, we studied expression of *GmNAC5* under ABA treatment (Figure 4(f)). The qRT-PCR assay suggested that expression of GmNAC5 was not markedly affected by ABA, suggesting that GmNAC5 may participate in ABA-independent signaling pathway in soybean under abiotic stresses.

## 4. Concluding Remarks

It has been documented that the plant-specific NAC (for NAM, ATAF1, 2, and CUC2) transcription factors play an important role in plant development and stress responses [27]. GmNAC5 belongs to the NAM subgroup and is most closely related to CUC1, CUC2, and NAM, which are involved in developmental events, maintenance of shoot meristem, and cotyledons separation [28]. In this study, we observed some new clues involved in the functions of GmNAC5. Tissue-specific expression analysis indicated that *GmNAC5* was highly expressed in immature seeds at 40 DAF and in the roots, suggesting the involvements of GmNAC5 in seed development and root development. It was also found that transcripts of *Arabidopsis AtNAC2* were accumulated at the late stages of seed development [29]. 

 It is also possible that higher expression of *GmNAC5* in soybean roots is associated with abiotic stress responses. *Arabidopsis* AtNAC2 expression was highly in roots and induced by salt stress [21]. Further studies suggested that AtNAC2 functioned downstream of ethylene and auxin signaling pathways and regulated lateral root development under salt stress. Expression of *GmNAC5* was significantly induced by multiple abiotic stresses but not by ABA, suggesting that GmNAC5 may be involved in ABA-independent stress responses in soybean under abiotic stresses. It was previously reported that NAC transcription factor involves the control of plant senescence and transient expression of *GmNAC5* in tobacco leaves induced senescence and necrosis, suggesting that GmNAC5 may play a role in the regulation of stress promoted senescence. Through microarray analysis, it was found that *Arabidopsis* AtNAC2 regulated many senescence-related genes and the majority of them are also regulated by salt stress, a major promoter of plant senescence [29]. Whether GmNAC5 plays a regulatory role in stress regulated root development or stress promoted senescence still needs to be further analyzed.

## References

[1] Mao X, Zhang H, Qian X, Li A, Zhao G, Jing R (2012). TaNAC2, a NAC-type wheat transcription factor conferring enhanced multiple abiotic stress tolerances in Arabidopsis. *Journal of Experimental Botany*.

[2] Xie Q, Frugis G, Colgan D, Chua N-H (2000). Arabidopsis NAC1 transduces auxin signal downstream of TIR1 to promote lateral root development. *Genes and Development*.

[3] Takada S, Hibara K-I, Ishida T, Tasaka M (2001). The CUP-SHAPED *COTYLEDON1* gene of Arabidopsis regulates shoot apical meristem formation. *Development*.

[4] Duval M, Hsieh T-F, Kim SY, Thomas TL (2002). Molecular characterization of AtNAM: a member of the Arabidopsis NAC domain superfamily. *Plant Molecular Biology*.

[5] Souer E, Van Houwelingen A, Kloos D, Mol J, Koes R (1996). The no apical Meristem gene of petunia is required for pattern formation in embryos and flowers and is expressed at meristem and primordia boundaries. *Cell*.

[6] Fujita M, Fujita Y, Maruyama K (2004). A dehydration-induced NAC protein, RD26, is involved in a novel ABA-dependent stress-signaling pathway. *Plant Journal*.

[7] Sablowski RWM, Meyerowitz EM (1998). A homolog of NO APICAL MERISTEM is an immediate target of the floral homeotic genes *APETALA3/PISTILLATA*. *Cell*.

[8] Iwase A, Hideno A, Watanabe K, Mitsuda N, Ohme-Takagi M (2009). A chimeric NST repressor has the potential to improve glucose productivity from plant cell walls. *Journal of Biotechnology*.

[9] Mitsuda N, Iwase A, Yamamoto H (2007). NAC transcription factors, NST1 and NST3, are key regulators of the formation of secondary walls in woody tissues of Arabidopsis. *Plant Cell*.

[10] Mitsuda N, Ohme-Takagi M (2008). NAC transcription factors NST1 and NST3 regulate pod shattering in a partially redundant manner by promoting secondary wall formation after the establishment of tissue identity. *Plant Journal*.

[11] Nogueira FTS, Schlögl PS, Camargo SR (2005). SsNAC23, a member of the NAC domain protein family, is associated with cold, herbivory and water stress in sugarcane. *Plant Science*.

[12] Ohnishi T, Sugahara S, Yamada T (2005). OsNAC6, a member of the NAC gene family, is induced by various stresses in rice. *Genes and Genetic Systems*.

[13] Hu H, Dai M, Yao J (2006). Overexpressing a NAM, ATAF, and CUC (NAC) transcription factor enhances drought resistance and salt tolerance in rice. *Proceedings of the National Academy of Sciences of the United States of America*.

[14] Han X, He G, Zhao S, Guo C, Lu M (2012). Expression analysis of two NAC transcription factors *PtNAC068* and *PtNAC154* from poplar. *Plant Molecular Biology Reporter*.

[15] Ooka H, Satoh K, Doi K (2003). Comprehensive analysis of NAC family genes in *Oryza sativa* and *Arabidopsis thaliana*. *DNA Research*.

[16] Taoka K-I, Yanagimoto Y, Daimon Y, Hibara K-I, Aida M, Tasaka M (2004). The NAC domain mediates functional specificity of CUP-SHAPED COTYLEDON proteins. *Plant Journal*.

[17] Hibara K-I, Takada S, Tasaka M (2003). CUC1 gene activates the expression of SAM-related genes to induce adventitious shoot formation. *Plant Journal*.

[18] Hasson A, Plessis A, Blein T (2011). Evolution and diverse roles of the CUP-SHAPED COTYLEDON genes in Arabidopsis leaf development. *Plant Cell*.

[19] Weir I, Lu J, Cook H, Causier B, Schwarz-Sommer Z, Davies B (2004). Cupuliformis establishes lateral organ boundaries in *Antirrhinum*. *Development*.

[20] Ruiz-Medrano R, Xoconostle-Cázares B, Lucas WJ (1999). Phloem long-distance transport of CmNACP mRNA: implications for supracellular regulation in plants. *Development*.

[21] He X-J, Mu R-L, Cao W-H, Zhang Z-G, Zhang J-S, Chen S-Y (2005). AtNAC2, a transcription factor downstream of ethylene and auxin signaling pathways, is involved in salt stress response and lateral root development. *Plant Journal*.

[22] Meng Q, Zhang C, Gai J, Yu D (2007). Molecular cloning, sequence characterization and tissue-specific expression of six NAC-like genes in soybean (*Glycine max* (L.) Merr.). *Journal of Plant Physiology*.

[23] Fehr WR, Caviness CE, Burmood DT, Pennington JS (1971). Stage of development descriptions for soybeans, *Glycine max* (L.) Merrill. *Crop Science*.

[24] Huang F, Chi Y, Gai J, Yu D (2009). Identification of transcription factors predominantly expressed in soybean flowers and characterization of *GmSEP1* encoding a SEPALLATA1-like protein. *Gene*.

[25] Ernst HA, Olsen AN, Skriver K, Larsen S, Lo Leggio L (2004). Structure of the conserved domain of ANAC, a member of the NAC family of transcription factors. *EMBO Reports*.

[26] Tran L-SP, Nakashima K, Sakuma Y (2004). Isolation and functional analysis of arabidopsis stress-inducible NAC transcription factors that bind to a drought-responsive *cis*-element in the early responsive to dehydration stress 1 promoter. *Plant Cell*.

[27] Peng H, Cheng H-Y, Yu X-W (2009). Characterization of a chickpea (*Cicer arietinum* L.) NAC family gene, CarNAC5, which is both developmentally- and stress-regulated. *Plant Physiology and Biochemistry*.

[28] Olsen AN, Ernst HA, Leggio LL, Skriver K (2005). NAC transcription factors: structurally distinct, functionally diverse. *Trends in Plant Science*.

[29] Balazadeh S, Siddiqui H, Allu AD (2010). A gene regulatory network controlled by the NAC transcription factor ANAC092/AtNAC2/ORE1 during salt-promoted senescence. *Plant Journal*.

